# Lateral Inhibition in the Human Visual System in Patients with Glaucoma and Healthy Subjects: A Case-Control Study

**DOI:** 10.1371/journal.pone.0151006

**Published:** 2016-03-08

**Authors:** Francisco G. Junoy Montolio, Wilma Meems, Marieke S. A. Janssens, Lucas Stam, Nomdo M. Jansonius

**Affiliations:** 1 Dept. of Ophthalmology, University Medical Center Groningen, University of Groningen, Groningen, the Netherlands; 2 Dept. of Epidemiology, Erasmus Medical Center, Rotterdam, the Netherlands; State University of New York Downstate Medical Center, UNITED STATES

## Abstract

In glaucoma, the density of retinal ganglion cells is reduced. It is largely unknown how this influences retinal information processing. An increase in spatial summation and a decrease in contrast gain control and contrast adaptation have been reported. A decrease in lateral inhibition might also arise. This could result in a larger than expected response to some stimuli, which could mask ganglion cell loss on functional testing (structure-function discrepancy). The aim of this study was to compare lateral inhibition between glaucoma patients and healthy subjects; we used a case-control design. Cases (n = 18) were selected to have advanced visual field loss in combination with a normal visual acuity. Controls (n = 50) were not allowed to have symptoms or signs of any eye disease. Lateral inhibition was measured psychophysically on a computer screen, with (1) a modified illusory movement experiment and (2) a contrast sensitivity (CS) test. Illusory movement was quantified by nulling it with a real movement; measure of lateral inhibition was the amount of illusory movement. CS was measured at 1 and 4 cycles per degree (cpd); measure of lateral inhibition was the difference between log CS at 4 and 1 cpd. Both measures were compared between cases and controls; analyses were adjusted for age and gender. There was no difference between cases and controls for these two measures of lateral inhibition (*p* = 0.58 for illusory movement; *p* = 0.20 for CS). The movement threshold was higher in cases than in controls (*p* = 0.008) and log CS was lower, at both 1 (-0.20; *p* = 0.008) and 4 (-0.28; *p* = 0.001) cpd. Our results indicate that spatially antagonistic mechanisms are not specifically affected in glaucoma, at least not in the intact center of a severely damaged visual field. This suggests that the structure-function discrepancy in glaucoma is not related to a decrease in lateral inhibition.

## Introduction

Glaucoma is a chronic and progressive eye disease characterized by loss of retinal ganglion cells (RGCs) and subsequent visual field loss. It is largely unknown how the loss of RGCs influences information processing within the retina. An increase in Ricco's area has been described [1] as well as changes in contrast gain control and contrast adaptation (see Discussion section). A decrease in lateral inhibition might also arise [2]. This could result in a larger than expected response to some stimuli, which could mask RGC loss on functional glaucoma testing. A decrease in lateral inhibition may thus play a role in the presumed observation that structural loss precedes functional loss in glaucoma (structure-function discrepancy).

The aim of this study was to compare lateral inhibition in the visual system between glaucoma patients and healthy subjects. For this purpose we developed a new psychophysical method and applied this method to glaucoma patients and controls. We also performed contrast sensitivity (CS) measurements at 1 and 4 cycles per degree (cpd). The difference between the CS at 4 and 1 cpd is presumed to be a measure of lateral inhibition as well (the inhibition makes the visual system optimally tuned for spatial frequencies around 4 cpd—for photopic vision in the center of the visual field) [3,4].

## Materials and Methods

### Study population

The present study was a case-control study and comprised 18 glaucoma patients (cases) and 50 healthy subjects (controls). The ethics board of the University Medical Center Groningen (UMCG) approved the study protocol. All participants provided written informed consent. The study followed the tenets of the Declaration of Helsinki.

Glaucoma patients were selected from visitors of the outpatient department of the department of Ophthalmology, UMCG, using the visual field database of the Groningen Longitudinal Glaucoma Study (GLGS), a prospective observational cohort study performed in a clinical setting [5]. The subpopulation selected for the present study comprised open angle glaucoma patients (primary n = 16, pigment dispersion n = 1, pseudoexfoliation n = 1) with (1) a visual field mean deviation (MD) of -12 dB or worse (as measured with standard automated perimetry [Humphrey Field Analyzer 30–2 SITA fast; Carl Zeiss, Jena, Germany]) and (2) a best-corrected visual acuity (BCVA) of 0.0 logMAR or better (up to 50 years of age) or 0.1 logMAR or better (above 50 years), in at least one eye. If both eyes met the inclusion criteria, the eye with the lowest MD value was chosen.

Healthy subjects were recruited by advertisement. We aimed for subjects between 40 and 70 years of age, at least 15 subjects per decade, with a ratio of approximately 3 controls per case. First, healthy volunteers who responded to the advertisement were asked to complete a questionnaire to screen for any known eye abnormality and a positive family history of glaucoma (exclusion criteria). After this preselection, the subjects completed an eye examination, including a BCVA measurement, a non-contact intraocular pressure (IOP) measurement (TCT80; Topcon Medical Systems, Oakland, USA), and a fundus examination with the Optos ultra-widefield retinal imaging device (200TX; Optos, Marlborough, USA). Exclusion criteria consisted of any known eye abnormality, a positive family history of glaucoma, a BCVA worse than 0.0 logMAR (up to 50 years of age) or 0.1 logMAR (above 50 years), an IOP above 21 mmHg, a vertical cup-to-disc ratio above 0.7 [6] or any other fundus abnormality (as observed by an ophthalmologist [NJ] who evaluated the Optos images and all other available data; in case of doubt, the subject was re-invited and a full eye exam was performed including fundoscopy in mydriasis as well as laser polarimetry of the optic nerve head (GDx ECC; Carl Zeiss, Jena, Germany) and a frequency doubling technology visual field test (FDT C20-1 screening mode; Carl Zeiss, Jena, Germany). A GDx VFI above 35 or any reproducibly abnormal test location at *p*<0.01 on the FDT test result implied exclusion. If both eyes were eligible, the dominant eye was chosen according to the Dolman method [7].

### Lateral inhibition

Two different psychophysical experiments were performed. Both experiments target spatially antagonistic mechanisms, of which the physiological equivalent is presumed to be lateral inhibition (see Discussion section). The experiments were carried out monocularly, in a sparsely illuminated room (luminance of the wall typically 10 cd/m^2^; luminance of the screen (see below) if switched off < 1 cd/m^2^). No cycloplegia, mydriasis, or artificial pupil was used. All experiments were performed with optimal correction for the viewing distance. Pupil diameter was measured with a ruler, while the subject was looking at the stimulus with the contralateral eye occluded (as was the case during the experiments).

#### Illusory movement

**Fig 1:**
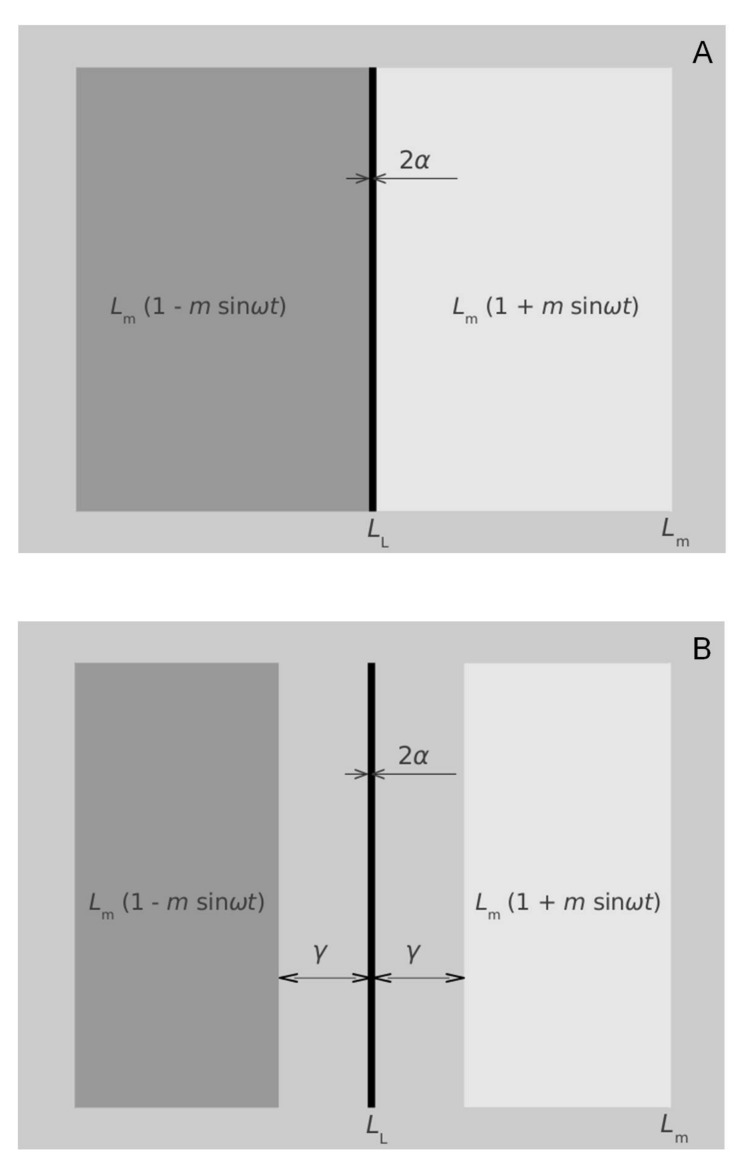
**Original (A) and modified (B) illusory movement stimulus**. 2*α* is the line width, *L*_m_ the mean luminance, *L*_L_ the luminance of the line, *m* the modulation depth, *ω*/2π the modulation frequency, and *γ* the distance between the line and the border of the stripes.

The first experiment is based on a psychophysical phenomenon called illusory movement. Illusory movement has been described in detail by Jansonius et al. [7]. In short, a narrow bar or line (width around 1 arcminute) between two fields of which the luminances are sinusoidally and in counterphase modulated in time (Fig 1A) appears to make an oscillatory movement. It is possible to annihilate this illusory movement with a real movement and thus to analyze this phenomenon quantitatively. The phenomenon can be explained by a model that includes low-pass spatial filtering in the visual system. With some modification, that is, replacing the modulated fields by modulated stripes positioned at a certain distance from the line (Fig 1B), a 180 degree phase shift occurs and this shift is presumed to reflect lateral inhibition [8,9]. Following Kooi and Kuiper [8] and Jansonius and Kuiper [9], we used a line width of 1.2 arcminute and a distance between the line and the border of the stripes of 6 arcminute. Modulation depth was 0.08; modulation frequency 2.5 Hz.

The stimulus was generated on an LCD monitor (Samsung SyncMaster 2243WM). The refresh rate was 75 Hz; the mean luminance of the screen 150 cd/m^2^ as measured with a Minolta luminance meter with built-in photometric filter (LS-110; Minolta Camera Co. Ltd., Japan). The refresh rate is far beyond the critical fusion frequency, which is about 40 Hz at this luminance [10,11]. The stimulus was generated and the data were collected using Octave (version 3.2.4; www.gnu.org/software/octave/) for Linux (Ubuntu 10.10) in combination with the Psychophysics Toolbox (PTB-3) [12,13]. The distance between subject and screen was 6 m. The screen size was about 4 degrees (0.4 m at 6 m); the stimulus size was limited by a digital mask to 1 by 1 degree. The luminances of the area outside the mask, the areas between the line and the stripes, and the mean luminances of the modulated stripes were equal (150 cd/m^2^).

**Fig 2:**
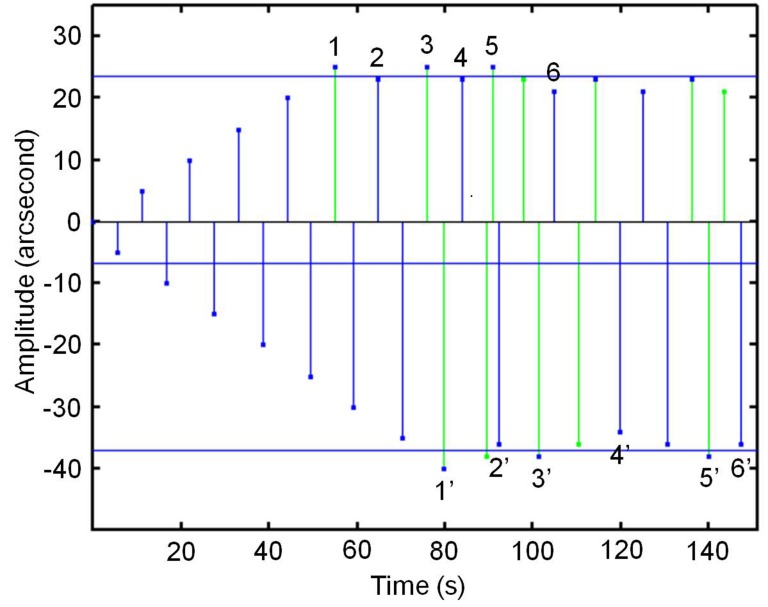
Staircase method applied to the illusory movement experiment.

We measured the illusory movement by compensating it with a real movement, using a staircase method. Fig 2 illustrates this method. The stimulus is presented during 4 s and the subject has to report yes/no movement observed. In the beginning, no real movement is added (in this situation, no movement is observed because the threshold for seeing movement is larger than the illusory movement itself). Subsequently, real movement is added in steps of 5 arcsecond (amplitude of the oscillatory movement) until movement is observed, subsequently removed in steps of 2 arcsecond until no movement is observed, added in steps of 2 arcsecond until movement is observed, and so on. In this way, six reversals are collected. Another six reversals are collected by making the initial 5 arcsecond steps in the opposite direction. These two series of reversals are collected alternately (Fig 2). From these two series of reversals, we calculated two thresholds: an overcompensation threshold and an undercompensation threshold. For each threshold we calculated two median values from the corresponding reversals (see Fig 2: median of 1,3,5 and median of 2,4,6 for the upper threshold; median of 1',3',5' and median of 2',4',6' for the lower threshold) and averaged the two medians. Two outcome measures were subsequently calculated: (1) the amount of illusory movement (the measure of lateral inhibition), which is the average of the overcompensation threshold and the undercompensation threshold and (2) the movement threshold (the difference threshold between illusory movement and real movement), which is the difference between the overcompensation and undercompensation threshold divided by two.

The experiment was performed three times, preceded by a short try-out. The median test result of these three experiments was the final test result.

#### Contrast sensitivity

The second experiment consisted of a sine-wave grating CS test, using two spatial frequencies: 1 and 4 cpd. Here, the psychophysical method was a tracking method according to von Békésy [14,15]. At the beginning of the experiment, contrast is negligible and gradually increases. If the subject observes the sine-wave grating, a button is pushed and held, resulting in a gradually decrease in contrast. If the grating disappears, the button is released and the contrast increases again, and so on. Contrast changed with a speed of 0.3 log/s and a contrast threshold was calculated from 12 (6 upper and 6 lower) reversals. For details see Nio et al. [16]. Contrast sensitivity is the reciprocal of the contrast threshold. Contrast is defined as (*L*_max_-*L*_min_)/(*L*_max_+*L*_min_), where *L*_max_ and *L*_min_ are the maximum and minimum luminance on the screen, respectively. Hardware and software were identical to that of the illusory movement experiment; the mean luminance of the screen was 150 cd/m^2^. Testing distance was 4 m. The sine-wave gratings, which were oriented vertically, filled the entire screen, resulting in a stimulus size of approximately 6 (horizontally) by 4 (vertically) degrees.

The experiment was performed two times per spatial frequency, preceded by a short try-out. The mean value per spatial frequency was the final CS test result.

### Statistical analysis

Nonparametric descriptive statistics (median with interquartile range [IQR]) was used to describe the study population; the corresponding univariable comparisons between cases and controls were made with a Mann-Whitney test. Proportions were compared with the Chi-square test. Yates (continuity) correction was applied if applicable. Multiple linear regression was used to compare the outcome measures between cases and controls adjusted for age and gender. All analyzes were performed using R (version 2.11.1; R Foundation for Statistical Computing, Vienna, Austria). A *p* value of 0.05 or less was considered statistically significant. The data underlying this study are available as supplementary material (S1 File).

## Results

Table 1 presents the general characteristics of the study population. Glaucoma patients were older (*p*<0.001) and more often male (*p* = 0.012) compared to the healthy subjects. Visual acuity was statistically lower (logMAR higher) in the glaucoma patients (median [IQR] logMAR 0.00 [0.00 to 0.05]) than in the healthy subjects (-0.08 [-0.08 to 0.00]; *p*<0.001). The mean difference was 0.07. The difference became smaller after adjustment for age (0.05; *p* = 0.02; a logMAR difference of 0.05 corresponds to half a Snellen line). The glaucoma patients had a median (IQR) visual field MD of -23.5 (-26.9 to -17.2) dB. The pupil diameter did not differ between the groups (*p* = 0.16; *p* = 0.41 when adjusted for age; based on 14 cases and 17 controls for whom pupil diameter data were available).

**Table 1:** General characteristics of the study population.

	Cases (n = 18)	Controls (n = 50)	*p* value
Age (year; median [IQR])	70 (67 to 72)	55 (47 to 62)	<0.001
Gender (% female)	28	66	0.012
Pupil diameter (mm; median [IQR])*	4.3 (4.0 to 5.3)	5.0 (4.0 to 6.0)	0.16
Visual acuity (logMAR; median [IQR])	0.00 (0.00 to 0.05	-0.08 (-0.08 to 0.00)	<0.001

* = based on a subset of 14 cases and 17 controls with pupil-diameter data; IQR = interquartile range.

**Fig 3:**
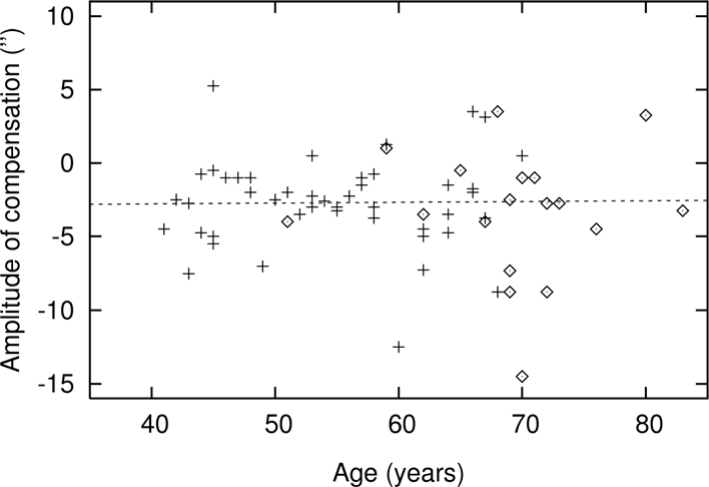
Amount of illusory movement as a function of age, for cases (◊) and controls (+). Regression line refers to the controls.

Fig 3 presents the results of the illusory movement experiment. There was no significant difference between cases and controls in a univariable analysis (*p* = 0.61) with a median (IQR) of -3.0 (-4.4 to -1.0) and -2.6 (-4.3 to -1.0) arcsecond for the cases and controls, respectively. Table 2 shows the results of the multivariable analysis. No significant difference between cases and controls was found when adjusted for age and gender (*p* = 0.58).

**Table 2:** Multivariable regression analysis showing the lateral inhibition outcome measures (amount of illusory movement and contrast sensitivity difference between 4 and 1 cpd), the movement threshold, and the contrast sensitivity test results, adjusted for age and gender.

		Beta	*p* value
Illusory movement*	Glaucoma	-0.740	0.58
	Age	0.005	0.93
	Gender	0.093	0.92
Movement threshold*	Glaucoma	13.820	0.008
	Age	0.208	0.33
	Gender	8.037	0.03
CS4-CS1	Glaucoma	-0.079	0.20
	Age	-0.004	0.11
	Gender	-0.045	0.31
CS1	Glaucoma	-0.204	0.008
	Age	0.002	0.60
	Gender	-0.023	0.67
CS4	Glaucoma	-0.283	0.001
	Age	-0.003	0.48
	Gender	-0.068	0.27

* = based on 18 cases and 46 controls (not all participants were able to perform this test); CS1 = contrast sensitivity measured at 1 cpd; CS4 = contrast sensitivity measured at 4 cpd.

**Fig 4:**
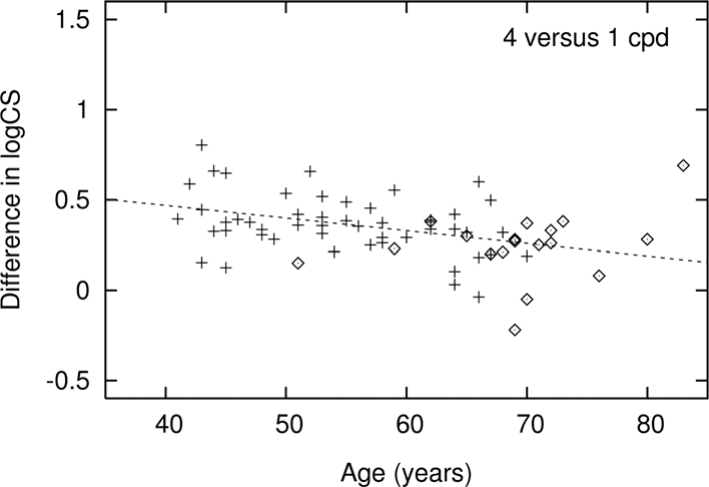
Difference in contrast sensitivity between 4 and 1 cpd as a function of age, for cases (◊) and controls (+). Regression line refers to the controls.

Fig 4 presents the results of the second experiment, the difference between the CS at 4 and 1 cpd. This experiment showed—in the univariable analysis—a significantly lower lateral inhibition in the glaucoma patients compared to the healthy subjects (median [IQR] 0.27 [0.20 to 0.32] versus 0.36 [0.29 to 0.44]; *p* = 0.006). Table 2 shows the results of the corresponding multivariable analysis. No significant difference between cases and controls was found when adjusted for age and gender (*p* = 0.20).

**Fig 5:**
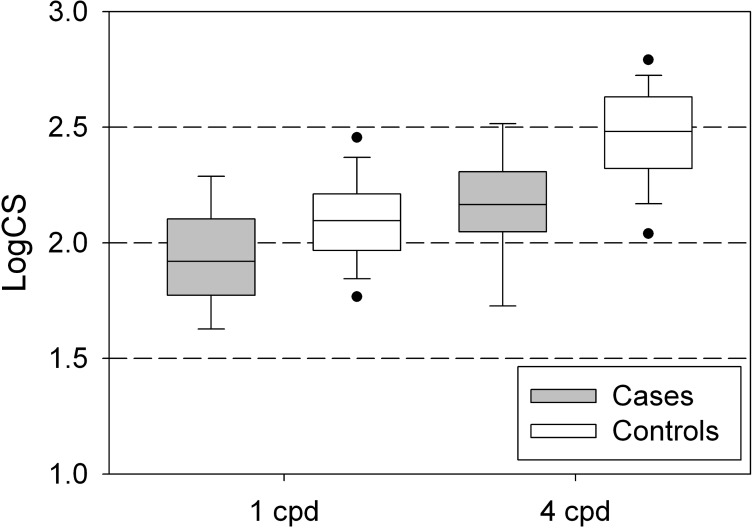
Contrast sensitivity at 1 and 4 cpd, for cases and controls (percentiles: 5, 10, 25, 50, 75, 90, and 95).

Fig 5 presents the underlying CS measurements at 1 and 4 cpd, for the cases and controls. Cases had a statistically significantly lower CS compared to controls, both at 1 (*p* = 0.008) and at 4 (*p*<0.001) cpd. These differences remained equally significant when adjusted for age and gender (Table 2).

The movement threshold (that is, the difference threshold between illusory movement and real movement) was larger in the glaucoma patients (median [IQR] 46 [31-60] arcsecond) than in the controls (30 [23-39] arcsecond; *p* = 0.003). A significant difference was also found in the multivariable analysis (Table 2).

## Discussion

Spatially antagonistic mechanisms are not specifically affected in glaucoma, at least not in the center of the visual field of patients with severe glaucoma and a normal visual acuity. The CS of the included patients was approximately half as high as the CS of the controls. Their movement threshold was significantly increased.

We found a single description of a decrease in lateral inhibition in glaucoma in the literature [2]. Sunga and Enoch used an experimental method as described by Westheimer [17]. They performed measurements both within relative scotomata and in apparently normal areas of the visual field (at an eccentricity of at least 5 degrees), in four patients with glaucomatous visual field loss. They found more lateral inhibition in the unaffected parts of the visual field than in the scotomata and hypothesized a retrograde damage of the synapses involved in lateral inhibition. We performed the measurements exclusively in an apparently unaffected area of the visual field. As such, our findings are in agreement with that of Sunga and Enoch. We focused on an unaffected area because our hypothesis (see Introduction section) was that the visual field would appear normal because an actual decrease in sensitivity was masked by a decrease in lateral inhibition. Given the glaucoma stage of the included patients, a significant thinning of the RGC layer is to be expected in the investigated area of the retina—despite the apparently unaffected function. Twelve of the 18 cases had had an assessment of the combined RGC layer/retinal nerve fiber layer/inner plexiform layer thickness in the macular area (6x6 mm scan centered around the fovea) with optical coherence tomography (OCT) as part of their regular glaucoma care. In 10 (83%) of them the thickness was outside normal limits according to the built-in normative database of the clinical device (Canon OCT HS-100; software version 1.0).

In his review paper on the control of sensitivity in the retina, Werblin described two types of lateral interactions [18]: antagonistic effects in stationary patterns mediated by horizontal cells (lateral inhibition in a narrow sense) and antagonistic effects in changing patterns mediated by amacrine cells. The former is related to predictive coding [19]; the latter to contrast gain control [20,21] and contrast adaptation [21,22]. Our stimuli were presumably targeting the former. For the—static—stimuli used to assess contrast sensitivity this is clear. The modulation used in the illusory movement experiment could theoretically trigger the contrast gain control mechanism as this mechanism already starts below our modulation frequency of 2.5 Hz; the applied modulation depth of 0.08, however, is much lower than used in psychophysical experiments targeting contrast gain control [23,24]. Moreover, as the illusory movement was assessed by nulling it with a real movement, contrast gain control should not influence it [7]. Contrast gain control and contrast adaptation have been shown to be affected in glaucoma [25–27]. Together with our results this suggests that glaucoma affects the inner retina more than the outer retina. However, there is some electrophysiological evidence for a generalized outer retina involvement in glaucoma [28] and histological changes in horizontal cells in humans with glaucoma have been described as well [29]. More recently, loss of horizontal cells has been described in mice [30]. This apparently contradicts earlier studies in the same animal [31,32].

The significant decrease in CS as found in this study is in line with earlier reports [33–35], although less clear effects have been published as well [36,37]. The observed decrease of 0.2 to 0.3 log units corresponds to almost a halving of the CS. Further, the movement threshold displayed a significant increase of approximately 50% (0.2 log units) in the glaucoma patients compared to the healthy subjects. A decrease in motion sensitivity has been described earlier, also in apparently normal areas of the visual field of glaucoma patients and in patients with ocular hypertension [38].

A normal lateral inhibition in glaucoma is not conflicting with an enlargement of the area of complete summation, also known as Ricco’s area, as recently described by Redmond et al. [1]. Due to lateral inhibition, mainly the RGCs at the edge of the stimulus contribute to the signal. This signal is reduced in glaucoma because of the loss of RGCs–the increase in Ricco's area, which implies an increase in the circumference (edge), compensates for this. If lateral inhibition would have been reduced, the RGCs in the center of the stimulus would contribute to the signal as well, which could initially mask glaucomatous RGC loss: our–falsified–hypothesis.

A limitation of this study is the difference in age distribution between the cases and the controls. Both test are subjective measurements and especially the illusory movement test is not easy to perform. For that reason we originally aimed to include subjects between 40 and 70 years of age. However, glaucoma is a disease of the elderly, and, as a consequence, the vast majority of the patients with severe glaucoma in our database was between 60 and 80 years old. This resulted in different age distributions in cases and controls, which hampered a direct comparison of the groups. However, the groups showed a considerable overlap in age and we adjusted all analyses for age. Also, none of the lateral inhibition measures showed a significant age dependency (Table 2). All this indicates that the different age distributions will not have influenced our major findings.

In conclusion, patients with severe glaucoma and a normal visual acuity and healthy controls display similar spatially antagonistic mechanisms in the central part of the visual field. Future research may focus on eccentric visual field areas, areas with a reduced sensitivity, and changing patterns.

## Supporting Information

S1 File Data underlying this study. (XLS)
